# Impact of Phone Call Intervention on Glycemic Control in Diabetes Patients: A Systematic Review and Meta-Analysis of Randomized, Controlled Trials

**DOI:** 10.1371/journal.pone.0089207

**Published:** 2014-02-19

**Authors:** Naeti Suksomboon, Nalinee Poolsup, Yuu Lay Nge

**Affiliations:** 1 Department of Pharmacy, Faculty of Pharmacy, Mahidol University, Bangkok, Thailand; 2 Department of Pharmacy, Faculty of Pharmacy, Silpakorn University, Nakhon-Pathom, Thailand; Universidad Peruana Cayetano Heredia, Peru

## Abstract

**Background:**

Telephone-delivered intervention can provide many supports in diabetes self-management to improve glycemic control. Several trials showed that telephone intervention was positively associated with glycemic outcomes in diabetes. The objective of this meta-analysis was to assess the impact of telephone contact intervention (intervention group) on glycemic control compared with standard clinical care (control group).

**Methods:**

Randomized control studies of telephone intervention in diabetes were searched on Medline (Pubmed), the Cochrane Central Register of Controlled Trials, Cumulative Index to Nursing and Allied Health Literature (CINAHL), Web of Science (ISI), and Scopus. Electronic search was done from inception to April 2013. The following MeSH terms were used: diabetes mellitus, randomized control trials and telemedicine, together with keywords including phone intervention, diabetes, and glycemic control. Historical search was also conducted on the references of relevant articles. The quality of the trials was assessed using Maastricht-Amsterdam scale. Treatment effect was estimated with mean difference in the change of hemoglobin A1c (HbA1c) from baseline between the intervention and control groups.

**Results:**

A total of 203 articles were examined. Five trials involving 953 patients met the inclusion criteria and contributed to the meta-analysis. Telephone contact intervention was no more effective than standard clinical care in improving glycemic control (pooled mean difference in HbA1c −0.38%, 95%CI −0.91 to 0.16%).

**Conclusions:**

This meta-analysis showed that the phone contact intervention was no more effective than standard clinical care in improving glycemic control in diabetes. However, telephone intervention may still have potential benefits especially for low-and middle-income countries; thus further large sample size and well-controlled studies are needed to evaluate the impact of the intervention.

## Introduction

Diabetes is a metabolic disorder that is characterized by hyperglycemia resulting from insulin deficiency, insulin resistance or both. The number of diabetes is increasing worldwide. There were 366 millions people with diabetes in 2011, 80% of which are from low- and middle-income countries (LMICs) [1]. For diabetes patients, there are many effective pharmacological and non-pharmacological treatments to improve glycemic control which is key to preventing serious macro- and micro-vascular complications [2]. Self-care activities are important factor to achieve targeted blood glucose levels, while outpatient follow-up is also needed for treatment success. However, many patients fail to achieve glycemic control because of inadequate out-patient services they receive in support of self-care management. They may also experience financial hardship that prevents them from regular follow-up assessment, especially in low-income countries [3], [4]. A lack of knowledge about diabetes and management skill also influences glycemic control in diabetes [5].

Telephone is widely used in the world and can be easily used by all age groups [6], [7]. International Telecommunication Union reported that the number of mobile-cellular telephone user is increasing worldwide although there was no significant increase in number of fixed-telephone users. In 2011, the number of fixed-telephone users was accounted for 542 millions in developed countries and 622 millions in developing countries. For mobile-cellular telephone, the number of users for developed and developing countries was 1475 and 4487 millions in 2011, and those in 2013 was likely to be 1600 and 5235 millions, respectively [8]. For more than 30 years, healthcare providers have been investigating the use of telemonitoring to improve clinical outcomes. The term “telemonitoring” is defined as the use of audio, video, and other telecommunication and electronic information processing technologies to monitor patient status at a distance [9]. Telephone support is one way of telemonitoring to give education related to disease and to support health consumers in self-management activities, such as medication adherence, physical exercise and diet [10]. With the use of mobile phone, many functions, such as short message service (SMS), photos, video and direct calls, internet access and software application support, can be used to help with patient self-activities [7]. Telephone monitoring saves time [11] and cost of transportation for patients living in long distance from healthcare center [3]. It can also overcome geographical problems [11], [12] or the difficulties facing elderly or disability patients [12]. Nonetheless, although telesupport may have positive effects in diabetes, phone intervention with sophisticated function may not be accessible for every patient, especially for patients with low socioeconomic status [6].

There were two qualitative systematic reviews of phone intervention in diabetes conducted by Holtz et al [10] and Krishma et al [13]. The results of both studies [10], [13] suggested that phone intervention may improve glycemic control in diabetes. One meta-analysis reported that mobile phone intervention decreased HbA1c by 0.5% [95% CI, 0.3%–0.7%] compared with control group over 6 months of intervention period [6]. Although these three reports [6], [10], [13] showed tendency for positive effects of telesupport intervention in diabetes, these kinds of intervention were based on modern devices and sophisticated technologies which cannot be applied in every developing country, especially for patients with low socioeconomic status. Although there was a systematic review of randomized controlled trials of phone calls intervention, it failed to pool the outcome data and lacked of strong evidence [14]. There has been no meta-analysis to support the evidence of phone contact intervention which can be applied to both developing and developed countries. This systematic review thus exclusively focused on direct phone call intervention. Phone call intervention with electronic data transmission to healthcare providers, phone call intervention together with the use of modern devices, such as glucometer and pedometer, or certain self-care activities that are not feasible in developing countries or could not be performed by all patient, such as self-monitoring of glucose and medication dose adjustment, are not considered in this review. We therefore aimed to assess the effectiveness of telephone call intervention compared with standard clinical care on glycemic control in diabetic patients.

## Methods

The protocol of this systematic review has not been registered.

### Literature Searches

A literature search for randomized control trials that evaluated the telephone intervention in diabetes was performed. An electronic searching was done on Medline (Pubmed), the Cochrane Central Register of Controlled Trials, Cumulative Index to Nursing and Allied Health Literature (CINAHL), Web of Science (ISI), and Scopus. The reference lists of potentially relevant trials, meta-analyses and systematic reviews were also searched. The electronic databases were searched from inception to April 2013. There was no language restriction. The MeSH terms used were diabetes mellitus, telephone, cellular phone, randomized controlled trial and telemedicine. The following keywords were also used; phone intervention, phone call, diabetes and glycemic control.

### Study Selection

Inclusion criteria for systematic review and meta-analysis included randomized control trial of phone contact intervention compared with standard clinical care in diabetes, and report of HbA1c as an outcome measure. We excluded studies that contained electronic transmission of outcomes data from patients to healthcare providers, enrolled gestational diabetes, were conducted in in-patients setting, utilized phone call intervention together with self-monitoring of blood glucose, medication adjustment by patients, or use of modern devices, such as pedometer and accelerometer. Two reviewers independently assessed and selected final eligible studies and disagreements were resolved by a third reviewer.

### Quality Assessment

Quality assessment was completed independently by two authors. Disagreements were resolved by a third investigator. Maastricht-Amsterdam scale [15] was used to assess the quality of the included studies. It contains 12 items to estimate the bias of the studies. The items have the ranking system of “yes,” “no,” or “unsure”. The item was ranked “yes” when the study met the criterion or “no” when the study did not meet the criteria. If there was no information for the item, it was ranked “unsure”. It can be assumed that the study is of low risk of bias when at least 6 criteria are met or of high risk of bias when fewer than 6 criteria are met.

### Data Extraction

Two authors independently extracted data from individual studies using standardized form, and any discrepancies were resolved by a third author. Extracted data included the characteristics of the patients, study characteristics, year of the publication, the country that the study was conducted, the frequency of phone calls, duration of each telephone session, and intervention components. The HbA1c levels in both groups were also extracted.

### Statistical Analysis

The change from baseline HbA1c (mean and standard deviation) in the intervention group and the control group was recorded. Treatment effect was estimated with mean difference in the change values between the two groups. When the variances of the change values were not provided, the pooled interstudy variance was imputed from studies reporting variances. The inverse variance-weighted method was used to pool mean difference and estimated 95% confidence interval [16]. Fixed effect model was used if the Chi square for the heterogeneity was not significant. If the heterogeneity was significant (P<0.1), random effect model was used. I^2^ statistic was used to estimate the variability among the included studies. I^2^ value of 0–40% might not be important, 30–60% may represent moderate heterogeneity, 50–90% may represent substantial heterogeneity, and 75–100% represents considerable heterogeneity [16]. Review Manger 5.2.3 (Cochrane Collaboration, Oxford, UK) was used to perform the statistical analysis and the significant level was set at P<0.05. Funnel plot and Egger’s method were performed to assess publication bias.

## Results

### Characteristics of the Included Studies

**Figure 1:**
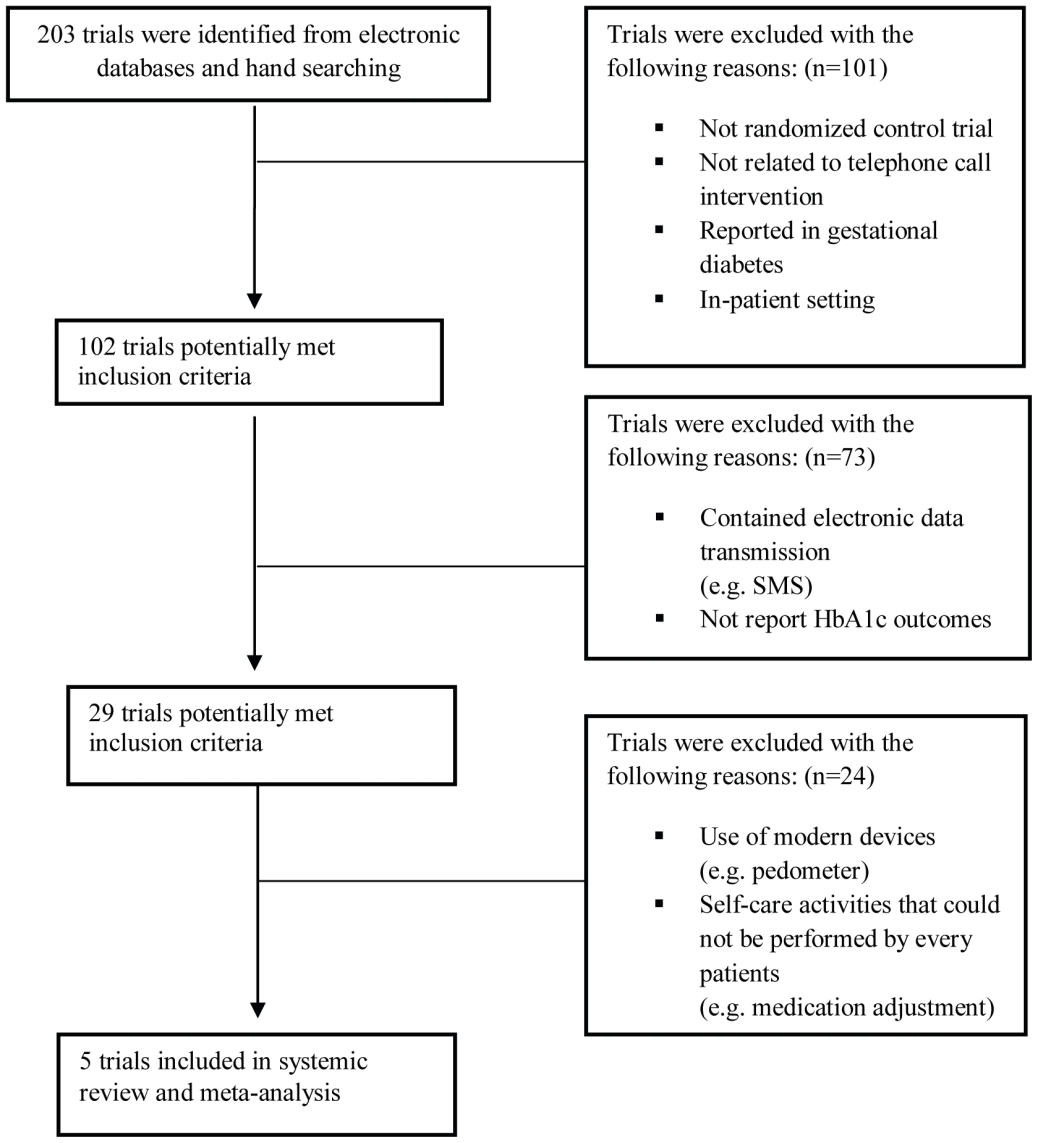
Flow of study selection for systematic review and meta-analysis.

Study selection process is presented in Figure 1. We initially identified 287 articles, of which 102 articles were screened. Twenty-nine studies potentially met the inclusion criteria. Of these, 24 studies were further excluded for the following reasons: they either used modern devices including accelerometer [17], log book [18], pedometer [19], [20] and glucometer [21], or the patients participating in the study employed self-care activities including self-glucose monitoring [5], [22]–[38] and medication adjustment by patients themselves [39]. Finally, there were five [40]–[44] studies that met the inclusion criteria and contributed to the meta-analysis.

The characteristics of the included studies are shown in Table 1. The number of patient in individual trials varied from 49 to 444. Two studies [41], [42] were three-armed randomized trials in which two intervention groups were compared with the control group. One trial [41] compared telephone support by peer supporter (group 1) or by diabetes nurse (group 2) against standard clinical care (group 3). Other study [42] compared two intervention arms with the control group. One intervention group received telesupport plus standard clinical care, while the other received telesupport plus standard clinical care and additional clinics visit. The results of each intervention arm were pooled and exclusively treated as the intervention group.

**Table 1:** Characteristics of the studies included in meta-analysis.

Study (ref)	Country	Quality score	N (Intervention: Control)	Subjects	Age (years)	Duration of diabetes (years)
Bogner 2012 [40]	USA	7	92∶ 88	T2DM	57.5±9.5	11.3±11.0
Dale 2009 [41]	UK	9	115∶ 86	T2DM	Not reported	Not reported
Howells 2002 [42]	UK	7	51∶ 28	T1DM	16.5	6.7
Walker 2011 [43]	USA	5	228∶ 216	T2DM	55.6±7.3	9.1±6.6
Whittemore 2004 [44]	USA	5	26∶ 23	T2DM	57.6±10.9	2.7±3.0

Data are mean ± SD or mean.

Table 2 summarizes the characteristics of the intervention and standard clinical care employed in individual studies. Both phone call intervention and standard clinical care greatly varied from trial to trial. For example, the number of phone calls ranged from 2 times to 16 times and each session took 9 minutes to 15 minutes. One study gave no details of standard clinical care while the other described standard clinical care that encompassed 3 or 4 monthly clinic visits and the patient also received the nurse-coaching intervention [44]. Details of study quality assessment are described in Table 3. Of the 5 studies, three studies [40]–[42] were of low risk of bias and two studies [43], [44] were of high risk of bias.

**Table 2:** Description of intervention and control in the included studies.

Study(ref)	Duration(months)	Intervention	Frequencyof calls	Call duration(minutes)	Intervention	Control
Bogner2012 [40]	3	Telephone callplus in-personcontact	2 calls	15	Education related to diabetes,depression and medication (oralhypoglycemic drugs andantidepressant), side effects and theirmanagement	Standard clinical care
Dale2000 [41]	6	Telephone call	4.5(average)1–6[range]	9.5 (average)1–37 [range**]**	Support to follow prescription ofphysician regularly, especially whentreatment changed	A single call during 3 or 5 days and encouragement to follow the treatment prescription
Howells2002 [42]	12	Telephone callplus in-personcontact	16 (average)5–19 [range]	9 (median)2–30 [range]	Standard clinical care plus defining theproblems and making solutions tosolve the problems and goal setting	Standard clinical care in which patients needed to visit clinic every 3 month and received diabetes education. Diabetes nurse also gave suggestion for problem solving by telephone, at hospital or home visits.
Walker2011 [43]	12	Telephone call	10 calls[4–6 weeklyinterval]	Notreported	Standard clinical care plus giving supportindividually depending on patients’ need,support to follow medication, clinic visitsand lifestyle modification, socialencouragement, and goal setting	Standard clinical care in which patients were provided with self-management materials.
Whittemore2004 [44]	6	Telephone callplus in-personcontact	2 calls	Notreported	Psychological support, educationabout diabetes, problem solving,and motivation	Patients needed to meet primary healthcare providers every 3–4 months together with nurse-coaching intervention at the end of the study

**Table 3:** Quality assessment of the included studies.

	Items	Bogner2012 [40]	Dale2000 [41]	Howells2002 [42]	Walker2011 [43]	Whittemor2004 [44]
1	Was the method of randomization adequate?	yes	yes	yes	yes	unsure
2	Was the treatment allocation concealed?	unsure	no	yes	unsure	unsure
3	Was the patient blinded to the intervention?	no	no	no	no	no
4	Was the care provider blinded to the intervention?	no	unsure	unsure	unsure	unsure
5	Was the outcome assessor blinded to the intervention?	yes	yes	unsure	unsure	unsure
6	Was the drop-out rate described and acceptable?	unsure	yes	unsure	unsure	yes
7	Were all randomized participants analyzed in the group to whichthey were allocated?	unsure	yes	unsure	yes	yes
8	Are reports of the study free of suggestion of selective outcome reporting?	yes	yes	yes	yes	yes
9	Were the groups similar at baseline regarding the most importantprognostic indicators?	yes	yes	yes	unsure	no
10	Were co-interventions avoided or similar?	yes	yes	yes	unsure	no
11	Was the compliance acceptable in all groups?	yes	yes	yes	yes	yes
12	Was the timing of the outcome assessment similar in all groups?	yes	yes	yes	yes	yes

### Efficacy

**Figure 2:**
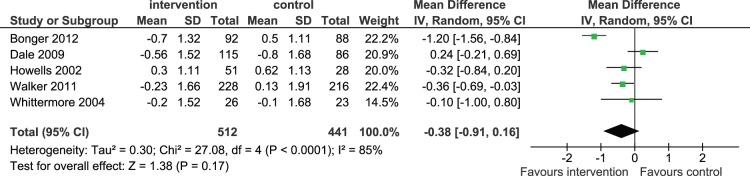
Mean difference (95% CI) in the changes of HbA1c from baseline for phone call intervention and standard clinical care.

**Figure 3:**
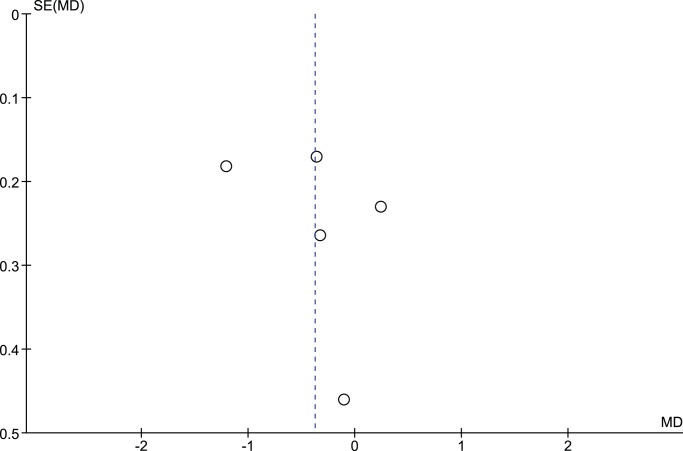
Funnel plot of the included studies.

Five randomized control trials [40]–[44] contributed to the meta-analysis. Telephone intervention did not significantly improve glycemic control as measured by HbA1c compared to standard clinical care (pooled mean difference - 0.38%, 95%CI - 0.91% to 0.16%) (Figure 2). Funnel plot and Egger’s method were performed and no publication bias was observed (Egger: bias = 2.97; 95% CI = −10.83 to 16.77, P = 0.54) (Figure 3).

## Discussion

This is the first study that evaluates phone intervention without electronic data in diabetes patient. Our study was designed to evaluate whether the phone contact intervention without electronic data transmission from patients to healthcare providers is effective in keeping glycemic control in diabetes patients. It was aimed to explore whether this intervention would be appropriate and can be considered for use in LMICs. Our meta-analysis included five trials and the result showed that telephone intervention did not significantly improve glycemic control compared to standard clinical care. Our results contrasted with previously published systematic reviews [6], [10], [13] in which the positive tendency of phone intervention was observed, however, they used modern devices to assess the patients’ outcomes therefore may have limited feasibility in LMICs.

It is noted that heterogeneity was significant among the included studies (I^2^ = 85%, P<0.0001). The differences in the characteristics of intervention, for example, frequency and duration of phone calls, components of intervention including information and psychological support provided, and variations in standard clinical care among individual trials, may have a role to play. Heterogeneity may also be attributable to differences in patient characteristics, especially the duration of diabetes. Patients in one of the included study [40] had been diagnosed with diabetes for over 10 years. In contrast, patients in other studies had less than 10 years of diabetes duration. Although the duration of diabetes does not necessarily directly affect glycemic control, it can influence the adherence to treatment regimen, which is strongly related to HbA1c outcomes. Patients with long duration of diabetes tend to reduce their interest and motivation to follow the self-care activities, such as poor medication adherence and lifestyle modification, compared with newly diagnosed patients [45], [46]. In addition, glycemic outcome is also affected by a number of other patient-related factors, such as age, education level, income, type of diabetes, and medication complexity [46].

Our meta-analysis showed no effectiveness of telephone monitoring without electronic data transmission in glycemic control. However, it may be effective in high-income countries because healthcare providers can use telephone monitoring alongside other intervention components available on high cost modern devices. To be more effective in telephone intervention, the supporters need to have good education and on-going training are required [41], [47]. Diabetes patients value more the suggestion by diabetes educators compared with peer supporters who were given training to provide the intervention. Healthcare providers should support interventions that are appropriate to the patient settings (urban or rural setting) and background because they may have different knowledge and economic level. The telesupporter should assess patients’conditions and complexity of management regimen and adjust patients’needs before giving intervention to them.

Our meta-analysis is not without limitations. First, it included only five trials with small number of patients. Second, significant heterogeneity existed. However, subgroup analysis was not possible due to the small number of study. This review also excluded gestational diabetes and, thus, our result may not be generalizable to this group of patients. Well-designed, large randomized controlled studies are warranted. Other outcome measures, such as fasting blood glucose level and patient satisfaction, are required to be further assessed. There is also a need to perform subgroup analysis depending on diabetes types, age, number of calls, and duration of phone call session.

## Conclusion

In conclusion, our meta-analysis showed that the phone contact intervention was no more effective than the standard clinical care in improving glycemic control in diabetes. However, because telephone intervention may still have potential benefits especially for LMICs, the impacts of the intervention in diabetes need to be further evaluated.

## Supporting Information

Checklist S1 **PRISMA 2009 Checklist.**
(DOCX)
